# State Medical Examinations

**Published:** 1895-11

**Authors:** William Warren Potter

**Affiliations:** Buffalo, N. Y., Member New York State Medical Examining and Licensing Board


### STATE MEDICAL EXAMINATIONS.

CONDUCTED BY WILLIAM WARREN POTTER, M. D., Buffalo, N. Y.,
Member New York State Medical Examining and Licensing Board.

### UNIVERSITY OF THE STATE OF NEW YORK MEDICAL EXAMINATIONS.

EXAMINATIONS for license to practise medicine in this state will be held as follows :

*Dates.*—1896 : January 28–31, April 7–10, May 19–22, June 16–19.

*Places.*—New York, Albany, Syracuse, Buffalo. Each candidate is notified as to exact place.

*Daily Program.*—Tuesday, morning, 9.15—12.15, anatomy ; afternoon, 1.15—4.15, physiology and hygiene. Wednesday, morning, 9.15—12.15, chemistry ; afternoon, 1.15—4.15, surgery. Thursday, morning, 9.15—12.15, obstetrics ; afternoon, 1.15—4.15, pathology and diagnosis. Friday, morning, 9.15—12.15, therapeutics.

---

### AN ANALYSIS OF THE WORK OF THE STATE MEDICAL EXAMINING AND LICENSING BOARD.

BY ALBERT T. LYTLE, M. D., Regent's examiner for Buffalo.

A MORE or less careful study of the percentages allowed by the examining board of the Medical Society of the State of New York, during the first three years of its existence, (academic years 1892, 1893 and 1894—the academic year begins August 1st and ends July 1st,) to candidates for license to practise medicine in this state, give some interesting facts that only too surely prove the wisdom of the step taken by the people and the advantages to be gained by a still greater demand upon those who wish to practise medicine in this great commonwealth. The high values of the following figures will, undoubtedly, be lowered as time passes, for, necessarily, the nature of the questions will call for more and more reasoning, deduction and comparison as the board gains experience and as the preliminary training of the applicants improves.

During the period just mentioned about one-third (33.99 per cent.) of all those who received the degree of Doctor of Medicine from a regular college in good standing in this state contested the examinations set for them by the board. Of the total number of applicants, sixty-five plus per cent. were from New York state colleges, nineteen plus per cent. were from the colleges of the other states and fourteen plus per cent. were from the colleges of foreign countries, *i. e.*, Canada, Mexico, England, and the like. Ninety-two (92.73) per cent. of those of New York, eighty-eight (88.65) per cent. of those of the other states and ninety (90.15) per cent. of the foreigners were granted licenses—not at all a bad showing for this state's institutions.

According to law, a candidate who fails is entitled to another trial after an interval of six months, in which he is expected to review the work ; also the number of examinations held yearly have been six, so that a candidate failing has been able to repeat at least once during the academic year. Now, the per cent. number of times that failures occurred as compared to the number of licenses granted was 10.71 for New York, 22.89 for the other states and 21.67 for the foreign countries, while the per cent. number of individuals failing, as compared with the number of licenses granted, was 9.35 for New York, 21.22 for the other states and 21.67 for the foreign countries. These figures seem to show that New York students had to repeat oftener than the other groups, or that when a candidate from one of the colleges out of New York state failed he sought to locate in fields easier of access, where the body-public was, probably, less sensitive as to qualifications. Every contestant who attains ninety per cent., or above, in six out of seven topics upon which questions are asked, receives an especial mark, called an "honor," which indicates a high degree of scholarship. This was granted to ten plus per cent. of the successful New York candidates, to fourteen plus per cent. of those from the other states and to less than two per cent. of those from foreign countries. As against foreign countries, New York makes a remarkable showing when is recalled the number of years of preparation demanded abroad, while comparison with other states is anything but flattering. Curiously, the highest individual average (99.7 per cent.) and the lowest that passed the board were both New York graduates. The general average per cent., for the whole period, for New York state candidates was 88.58 per cent., for those of the other states 90.44 per cent. and for those of foreign countries 88.39 per cent. In regard to the foreigners, allowance must be made for unfamiliarity with the English language, otherwise the best-equipped are not immigrating. As for the other states, it is evident that only those who are above the average in scholarly qualifications are endeavoring to settle here; consequently they will be of great advantage, not only professionally, but also in educating the people to be satisfied with only the best that the colleges can turn out. Briefly, these figures show that New York is harvesting a fair average crop and is importing a probably excellent one from abroad and an undoubtedly superior one from the sister states. This alone more than endorses the action of the state in taking into her own hands the determining of the especial fitness of those desiring to prevent and cure disease.

A careful study of the percentages in the different topics invariably shows better results in the three junior branches (anatomy, physiology, chemistry,) rather than in the four senior and more (?) important branches (surgery, obstetrics, pathology and diagnosis, therapeutics, practice and materia medica). It would seem that time enough is not given to the senior subjects, or, what is more probable, the methods of teaching are at fault; while, possibly, the nature of the questions in the junior group are too simple, or, what is more likely, the examiner unconsciously makes an allowance for their apparent secondary position in the study and the practice of medicine. The fault with the method of teaching is not peculiar to the senior subjects; its effect is but felt in a greater degree because therein reasoning, and not memory, is so largely required. Teaching in the primary and in the secondary schools, from which medical colleges secure the majority of their students, has been, until within a few years, practically one of exercising the memory, while reasoning, comparison and association have been left to the later coming college or life experience. As a large number of the medical teachers have had no better early training than that outlined, it is to be expected that, notwithstanding they have obtained their positions by their ability to reason and apply, yet, when it comes to the art of teaching, they follow in the "footsteps of their illustrious forefathers."

The work of the medical examining boards, through the efficient and thoroughly up-to-date management of the University of the State of New York, will improve the present methods of technical teaching by first supplying the institutions with better equipped material with which to work; by, secondly, compelling the technical teachers to prepare that material so that it will be able to stand the scrutiny of modern broadminded critics; and by, thirdly, so carefully guarding the fairness of all contests that the student will fully appreciate the inestimable value of the diplomas granted and of the honors awarded, as well as the high scholarship necessary to obtain them, and he will, therefore, demand instruction of the very highest possible degree of perfection that he may relatively easily obtain the coveted prizes.

